# 

**Published:** 1882-01

**Authors:** 


### INDEX TO VOLUME XXIX--1882.

A

| | |
|---|---|
| | PAGE. |
| Air, | 3 |
| Accidents, | 5 |
| About Dinner Hour, | 7 |
| An Iowa Girl's Ambition, | 11 |
| A Dangerous Curiosity, | 34 |
| A Sad Reflection, | 83 |
| An Orange Wrapping in Florida, | 105 |
| A Lesson in Etiquette, | 113 |
| A Small-Pox Preventive, | 160 |
| Animal Combustion, | 170 |
| A Clergyman's Congregation, | 172 |
| A Very Romantic History, | 179 |
| A Story of Abraham Lincoln, | 232 |
| A Machine that Measures Thought, | 238 |
| An Anecdote of Waterloo, | 278 |
| A Literary Curiosity, | 280 |
| A Happy Home, | 304 |
| Appetite, | 319 |
| Annual Ailments, | 339 |
| A Lay Sermon, | 348 |
| Autumnal Diseases, | 403 |
| Apples, | 450 |
| Africa's Population, | 504 |
| Arctic Mosquitoes, | 507 |
| About Bees, | 511 |
| A Strange Country, | 517 |

B

| | |
|---|---|
| Baths and Bathing, | 36 |
| Bad Handwriting, | 116 |
| Burial Customs on the Continent, | 121 |
| Be Systematic, | 223 |
| Beauty's Tyranny, | 228 |
| Bravery, | 462 |
| Bequests to Children, | 392 |
| Bad Teeth and Disease, | 528 |

**C**

Consumption Cure, - - - - - - 25
Consumption, - - - - - - 85, 183, 435
Cure for Drunkenness, - - - - - - 112
Cause of Death, - - - - - - 123
Cholera, - - - - - - - 149
Catching Cold, - - - - - - 168
Checking Perspiration, - - - - - 197
Cold Bathing, - - - - - - 204
Chinese Pirates, - - - - - 275
Care of Infants, - - - - - - 283
Croup, - - - - - - - 284
Can Animals Talk? - - - - - - 289
Coughing, - - - - - - 326
Civilization and Health, - - - - - 354
Climate for Consumptives, - - - - 338
Care of Children, - - - - - - 345
Cold in the Head, - - - - - 407
Cheapest Food, - - - - - - 383
Care of the Eyes, - - - - - 460
Checking Perspiration, - - - - - 490

**D**

Deterioration of the Eye, - - - - - 8
Drinking Water, - - - - - - 41
Diphtheria and Its Cause, - - - - 108
Diet of the Japanese, - - - - - 59
Death's Wèapons, - - - - - 153
Daniel Webster's Presence, - - - - - 164
Drinking at Meals, - - - - - 187
Dangers of Spring, - - - - - - 241
Decision of Character, - - - - - 320
Disease and Benevolence, - - - - - 360
Dirt, - - - - - - - 407
Digestion, - - - - - - - 467

**E**

Eight to Sixteen, - - - - - - 28
Exercise, - - - - - - - 218
English as the Speech of the Future, - - - - 237
Earliest Signs of Consumption, - - - - 324
Equanimity of Mind, - - - - - - 370
Eating and Drinking, - - - - - 428

**F**

Foods and How to Use Them, - - - - - 1
Farmers and Citizens, - - - - - - 29
Fever and Ague, - - - - - - 144
Feeding Horses, - - - - - - 351
Fishing on the Amazon, - - - - - 165
Facts Worth Remembering, - - - - - 346

Feeding the Sick, 405
Finger Nails, 394
Face Ache, 491
For the Farm and Family, 44
Facts for the People, 459

G

Going It, 33
Gossip, 63
Gratuitous Medical Advice, 200
Goethe and Schiller, 294
Great Things, 503
Germs of Disease, 506

H

Hygiene, 477
Hereditary Disease, 21
How People Take Cold, 77
Hot Air Furnaces, 98
How to Behave at the Table, 111
Heart Disease, 133
Health and Housekeeping, 141
How to Travel Cheaply, 158
How Silks Are Given Weight, 162
Health and Habit, 167
Health of Employments, 191
Households Contrasted, 208
Health for Children, 220
How to Exercise, 225
Hardships of Farmers' Wives, 245
How to Save a Drowning Person, 261
Health Maxims, 297
Health, 40, 327
Habit, 328
Heart Affections, 331
Human Teeth, 388
Hearty Suppers, 422
Health Capital, 345
Health of Employments, 483
How People Take Cold, 466
How Consumption May be Prevented, 498
House and Garden, 502
How to Mesmerize, 513
How Valuable Lives are Lost, 528

I

It Will Out, 227
India Rubber Hunting, 233
Ignorance and Ill Health, 249
Ice Water, 333
Intussusception, 358
Is Consumption Contagious? 408
Insanity, 361

Inhalation and Consumption, - - - - - 433
Influence of Temper on Health, - - - - - 379
Imprudence and Climate, - - - - - - 393
Influences, - - - - - - - - 402
Influence of Mind Over the Body - - - - 527

J

Japan, - - - - - - - - 155

K

Killing a Rhinoceros, - - - - - - 273
Kindness the Best Punishment, - - - - - 382

L

Life in the London Slums, - - - - - 61
Longevity, - - - - - - - 100
Laughter and Death (Poetry), - - - - 155
Laughing Horses, - - - - - - 177
Liquor Drinking, - - - - - - - 199
Life and Death, - - - - - - 297
Lead Colic, - - - - - - - - 306
Living Beyond One's Means, - - - - - 419
Longevity, - - - - - - - - 489
Labor and Capital, - - - - - - 505
Largest Cotton Planter in the World, - - - 516
Lincoln as a Peace-Maker, - - - - - 519

M

Midnight in Prison, - - - - - - - 14
Marvels of Man, - - - - - - - 37
My Life (Poetry), - - - - - - - 47
Mad Dogs, - - - - - - - - 55
Military Ants of the Amazon, - - - - - - 101
Men and Animals, - - - - - - - 107
Mind and Health, - - - - - - - 243
Moles, Warts, etc., - - - - - - 285
Mother and Infant, - - - - - - - 286
Medical Progress, - - - - - - - 322
Mental Rest, - - - - - - - - 425
Mind and Charcoal, - - - . - - 454
Mississippi Floods, - - - - - - - 500
Mining in Hot Places, - - - - - - 512

N

Norwegian Grave Yards, - - - - - 515
Nursing Animosities, - - - - - - - 394
Nutrition in Health and Disease, - - - - 530

O

Over Eating, - - - - - - - - 31
Out-Door Safety, - - - - - - - 329
Our Children's Teachers, - - - - - - 376

P

Pleasantly flavored, - - - - - - 12
Paris Crime, - - - - - - - - 13

Practical Knowledge, - - - - - - 26
Plants that Eat Animals - - - - - 60
Particles in the Eye, - - - - - - 176
Pure Food, - - - - - - - 208
Popular Fallacies, - - - - - - 215
Prevention of Disease, - - - - - - 231
Politics and Physique, - - - - - - 389
Paragraphs, - - - - - - - 265
Pneumonia, - - - - - - 343
Pure Water, - - - - - - - 353
Premature Deaths, - - - - 431
Prostitution of High Health, - - - - -
Perils of Flattery, - - - - - - 472
Poison Sumac, - - - - - - - 49
Poetry, - - - - - - - 476

Q

Quenching Thirst, - - - - - - 465

R

Recreation, - - - - - - - 39
Running up Stairs, - - - - - - 79
Reading, - - - - - - 97
Raising the Hat, - - - - - 177
Rheumatism, - - - - - - - 250
Reflections About Medical Men, - - - - 262
Reason in All Things, - - - - - - 272
Removal of Stains, - - - - - - 292
Restless Wanderers, - - - - - 373
Regimen, - - - - - - - 234
Remedy for Cough, - - - - - - - 397
Retiring from Business, - - - - - 443
Running up Stairs, - - - - - - 468

S

Small Pox, - - - - - - - - 23
Savages' Notions of Night-Mares, - - - - 117
Silo Experience, - - - - - - 119
Spirits, - - - - - - - - 125
Self Destroyers, - - - - - - - 130
Sleeping Together, - - - - - - 134
Spitting Blood, - - - - - - 193
Small Bed Chambers, - - - - - - 194
Sudden Death, - - - - - - 206
Sorrowing Poverty, - - - - - - 251
Shaving the Beard, - - - - - - 271
Sea Sickness, - - - - - - - 308
Sprains, - - - - - - - 330
Sleeping, - - - - - - - - 335
Scientific Notes, - - - - - - 350
Sea-Weed as an Anti-Fat, - - - - - 350
Slandering Doctors, - - - - - - 411
Servant Girls, - - - - - - - 356

Science Aiding Justice, - - - - - - 411
Style, - - - - - - - - 378
Science and Christianity, - - - - - - 414
Sleep, - - - - - - - - 392
Sunshine, - - - - - - - 428
Success in Life, - - - - - - 431
Sun Baths, - - - - - - - - 452
Sickness and Death, - - - - - - 462
Sugar and Teeth, - - - - - - 469
Snake Bites, - - - - - - - 496
Soap and Soap Making, - - - - - - 529

T

'Tis Easy to Die - - - - - - 9
The Sago Trees - - - - - - 10
The Teeth - - - - - - - - 15
The Nature of Consumption - - - - 21
The Feet in Winter - - - - - - 35
Taking Cold - - - - - - 57
The Horse on the Prairie - - - - - 62
The White Elephant - - - - - - 65
The Needle and Company - - - - - 67
The Dying - - - - - - - - 69
Tobacco - - - - - - - - 80
Their Jibbenenosey - - - - - 103
The Longevity of the Ancients - - - - 110
Trust (Poetry) - - - - - - - 114
Tomatoes - - - - - - - - 114
The Demon of Alcohol - - - - - - 115
To Stop Coughing - - - - - - - 128
The Instinct of Appetite - - - - - - - 135
The Achievements of Science - - - - - 138
The Story of the Tides - - - - - - 180
To Bring up Children - - - - - 189
Temperament Differences - - - - - 201
The Management of Sick Children - - - - 231
The Dining Hall in the 13th Century - - - 235
Traveling for Health - - - - - - 252
The Family Relation - - - - - - 259
The Coming Man, Physically - - - - - 290
The Pronunciation of "U" - - - - - 291
The Grave of Hope - - - - - - 383
The Teeth - - - - - - - 307
The Loss of Children - - - - - - 379
The Emotions - - - - - - - 318
Thermometers - - - - - - - - 377
Tobacco Users - - - - - - - 337
The Aim of Life - - - - - - - 374
Table Manners - - - - - - - 409
The Best Inheritance - - - - - - 415
Too White Flour - - - - - - - 443
Throat Ail - - - - - - - - 400

Tobacco - - - - - - - 460
Too Fat - - - - - - - 492
Tight Lacing - - - - - - 493
Typhoid Fever - - - - - - - 494
"Though Lost to Sight to Memory Dear" - - 497
To Stop Bleeding - - - - - - 497
The Use of Tobacco by Boys - - - - 501
The Timber Supply - - - - - - 509
Toothpicks - - - - - - 518
Tremors of the Earth - - - - - - 520
The Stinging Tree - - - - - - 520
Too Lean - - - - - - - 526

U

Unconscious reading - - - - - 274
Uncle Sam - - - - - - - 282

V

Ventilation - - - - - - - 169

Vermin Riddance - - - - - - 358

W

What is patent flour? - - - - - 174
Wearing the beard - - - - - - 214
Well done - - - - - - - 224
Wild animals and their tamers - - - - - 236
Why - - - - - - - 372
Washing - - - - - 404
Who are happiest - - - - 447

### BOOK NOTICES.

THE FATE OF MADAME LA TOUR.—A story of great Salt Lake, by Mrs. A. G. Paddock, is an exposé, in the form of an interesting story, of some of the crimes and outrages committed by the Mormons. The story shows what an influence the "latter-day saints" have over their subjects, and with what boldness they defy the laws of the country. It also gives a fair idea of life in a mining camp in the early days of the gold discoveries in California. Mrs. Paddock having resided in Salt Lake City for several years, has had every opportunity to observe the people of whom she writes. Published by Ford, Howard & Hulbert, New York.

PLOUGHED UNDER.—The story of an Indian chief, told by himself, in which he discusses the Indian question, and shows how wholly the red man is left to the mercy of the Indian agent. Ford, Howard & Hulbert, New York.

THE CHRISTIAN UNION for January 5th appears with a new and artistic heading, and the substitution of Roman for Italic titles and head-lines throughout the paper. The same number contains the article on the Utah problem, by the late Dr. Bacon, which was found unfinished on his desk the morning after his death. It treats the subject with the writer's accustomed force and with the pathos that attaches to anything that conveys one's last thoughts.

